# Supplementary material for: Short-Term Supplemental Dietary Potassium from Potato and Potassium Gluconate: Effect on Calcium Retention and Urinary pH in Pre-Hypertensive-to-Hypertensive Adults
Source: Nutrients. 2021 Dec 9;13(12):4399. doi: 10.3390/nu13124399 (PMC8707887; doi:10.3390/nu13124399)
Supplement: Supplementary file 1 [file nutrients-13-04399-s001.zip › nutrients-1476046-supplementary.pdf]

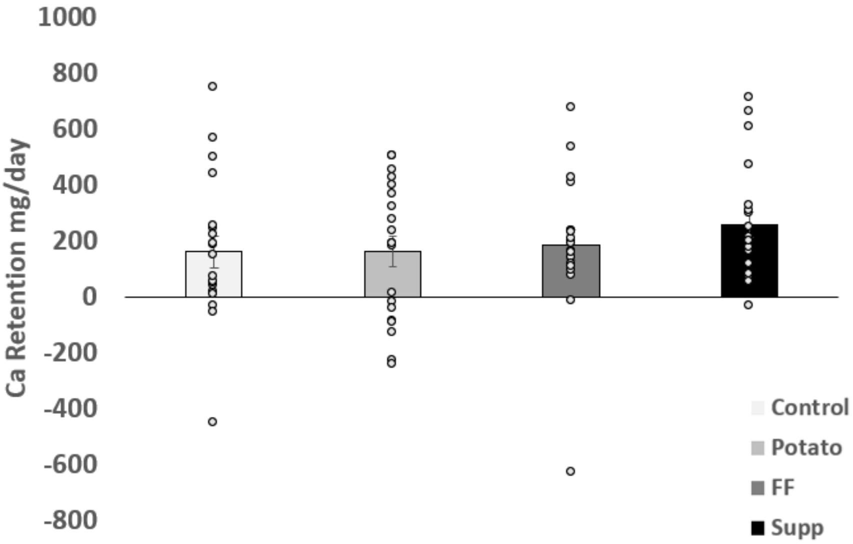

Figure S1. Means ± SE and individual data distribution of Calcium Retention (*n* = 20) as absolute calcium (Ca) retention. French fries (FF), or K gluconate (Supp).
